# TROP2-targeting antibody–drug conjugates in breast cancer and ovarian carcinoma: therapeutic advances and resistance mechanisms

**DOI:** 10.20517/cdr.2025.195

**Published:** 2026-02-25

**Authors:** Pilar Eroles, María Teresa Dawid de Vera, Víctor Lago

**Affiliations:** ^1^Department of Physiology, University of Valencia, Valencia 46010, Spain.; ^2^INCLIVA Biomedical Research Institute, Valencia 46010, Spain.; ^3^Center for Biomedical Network Research on Cancer (CIBERONC), Madrid 28019, Spain.; ^4^Health Research Institute Hospital La Fe (IISLAFE), Valencia 46026, Spain.; ^5^Department of Pathology, Hospital Universitari i Politecnic La Fe, Valencia 46026, Spain.; ^6^Department of Gynecologic Oncology, Hospital Universitari i Politecnic La Fe, Valencia 46026, Spain.; ^7^CEU Cardenal Herrera University, Moncada 46113, Spain.

**Keywords:** ADC resistance, TROP2, breast cancer, ovarian carcinoma

## Abstract

Antibody-drug conjugates (ADCs) targeting trophoblast cell-surface antigen 2 (TROP2) have emerged as a promising therapeutic strategy for the treatment of triple-negative breast cancer (TNBC) and ovarian carcinoma, two malignancies characterized by poor prognosis and limited therapeutic options. ADCs are complex molecules that combine the specificity of monoclonal antibodies with the cytotoxic potency of chemotherapeutic agents, enabling selective delivery of drugs to tumor cells while minimizing systemic toxicity. Recent advances in ADC technology have led to the development of several TROP2-targeting agents, including sacituzumab govitecan and datopotamab deruxtecan, which have demonstrated significant efficacy and acceptable safety in patients with advanced or treatment-resistant TNBC and ovarian carcinoma. Clinical trials have reported improvements in progression-free and overall survival, as well as objective response rates, compared to standard therapies. However, emerging evidence indicates that both primary and acquired resistance mechanisms may limit the long-term efficacy of these agents. Current research efforts are focused on elucidating these resistance pathways, optimizing combination strategies with immunotherapy and targeted agents, and expanding the application of TROP2-targeting ADCs to other tumor types. The integration of biomarker-driven patient selection and next-generation ADC technologies offers new opportunities to overcome resistance and enhance clinical benefit. This review provides a comprehensive overview of the development, clinical implementation, and resistance mechanisms of TROP2-targeting ADCs in TNBC and ovarian carcinoma, underscoring their potential to reshape the therapeutic landscape of these challenging cancers.

## INTRODUCTION

Antibody-drug conjugates (ADCs) constitute an innovative category of targeted cancer treatments that have markedly contributed to progress in oncology. By harnessing the specificity of monoclonal antibodies and the potent cytotoxicity of chemotherapeutic agents, ADCs are designed to selectively deliver cytotoxic payloads to tumor cells while minimizing damage to healthy tissues. This targeted approach is achieved through the conjugation of a monoclonal antibody, which recognizes and binds to tumor-associated antigens, to a cytotoxic drug via a specialized linker. The efficacy of ADCs is contingent upon the careful selection of tumor-specific antigens, the stability of the linker in systemic circulation, and the potency of the cytotoxic payload^[1]^.

Although originally designed for hematologic cancers, ADCs have quickly expanded their clinical applications to include various solid tumors. Notable milestones include the approval of agents such as gemtuzumab ozogamicin for acute myeloid leukemia^[1]^ and ado-trastuzumab emtansine for human epidermal growth factor receptor 2 positive (HER2+) metastatic breast cancer (BC)^[1]^. These successes have paved the way for the development of next-generation ADCs with improved selectivity, efficacy, and safety profiles.

Despite these advances, resistance to ADC therapy has emerged as a major barrier to sustained clinical benefit. Mechanisms of resistance can arise at multiple levels, including antigen-related factors (downregulation or heterogeneous expression of the target), defects in ADC internalization or trafficking, drug efflux mediated by transporters [e.g., adenosine triphosphate (ATP)-binding cassette (ABC) family proteins], altered linker stability, and changes in payload sensitivity due to enhanced DNA repair or apoptotic pathway modulation. Understanding these processes is essential for optimizing ADC design and guiding rational combination strategies.

Triple-negative breast cancer (TNBC) and ovarian carcinoma are among the most aggressive and treatment-resistant malignancies, characterized by poor prognosis and frequent relapse. TNBC, lacking estrogen (ER) and progesterone receptors (PR) and human epidermal growth factor receptor 2 (HER2) amplification, displays intrinsic chemoresistance and limited responsiveness to standard therapies. Likewise, ovarian carcinoma is commonly diagnosed at advanced stages, where resistance to platinum-based chemotherapy contributes to poor survival outcomes^[2,3]^. These challenges have prompted an intensified focus on novel targets and resistance-modulating therapeutic strategies, including ADCs^[4,5]^.

Trophoblast cell-surface antigen 2 (TROP2) has emerged as a compelling ADC target due to its high expression across multiple epithelial tumors. Overexpression of TROP2 correlates with tumor aggressiveness, metastasis, and poor clinical outcomes, making it both a prognostic biomarker and therapeutic target^[6]^. TROP2 is overexpressed in BC, with the highest prevalence observed in TNBC compared to other subtypes. Elevated TROP2 expression has also been reported in ovarian carcinoma. These findings highlight TROP2 as a highly promising target for the development of ADCs in these malignancies. Indeed, several TROP2-directed ADCs, such as sacituzumab govitecan (SG) and datopotamab deruxtecan (Dato-DXd), have demonstrated notable efficacy and manageable toxicity in patients with advanced or refractory disease. Nevertheless, the development of primary and acquired resistance to these agents remains a critical clinical challenge^[7]^.

Ongoing research is focused on overcoming ADC resistance through improved linker chemistry, alternative payloads with distinct mechanisms of action, modulation of antigen expression, and strategic combination with immunotherapy or DNA-damage response inhibitors. The integration of biomarker-driven patient selection, alongside next-generation ADC engineering, holds promise for enhancing therapeutic durability and expanding the clinical utility of TROP2-targeting ADCs in TNBC, ovarian carcinoma, and other solid tumors^[8-11]^.

## ADCS IN ONCOLOGY

ADCs are a group of novel cancer therapies that seek to deliver cytotoxic drugs via molecules selectively expressed in tumors. They combine three essential components: a monoclonal antibody, a cytotoxic payload, and a linker. The antibody must recognize a cell-surface antigen that is either exclusively expressed or highly overexpressed on malignant cells. The payload is usually a highly cytotoxic agent, effective at intracellular concentrations achievable within solid tumors, and potent enough to kill cancer cells, often requiring picomolar activity^[12]^. The linker connects the antibody and the cytotoxic drug. It must be stable in circulation to prevent premature drug release and be designed to release the active drug inside cancer cells, either through cleavable bonds or proteolytic degradation. Their success depends on selecting specific antigens unique to cancer cells to ensure precise targeting and minimize harm to normal tissue. Most ADCs use potent cytotoxic agents, such as microtubule inhibitors or DNA-damaging drugs, designed to induce apoptosis in cancer cells. ADCs can also induce a “bystander effect”, where released payloads permeate neighboring cells, enhancing anti-tumor activity and modifying the tumor microenvironment^[13]^.

ADCs were originally developed for hematologic cancers, where target antigens are well-defined, lineage-specific, and accessible on cell surfaces. The first U.S. Food and Drug Administration (FDA)-approved ADC in 2000 was gemtuzumab ozogamicin (Mylotarg), used for relapsed acute myeloid leukemia. Second-generation ADCs enhanced selectivity and minimized side effects. Notable approvals include brentuximab vedotin (2011) for treating Hodgkin lymphoma^[14]^ and inotuzumab ozogamicin, which targets CD22 on B cells and has shown promising results in relapsed/refractory acute lymphoblastic leukemia^[1]^.

In BC, the first major breakthrough in ADCs came with ado-trastuzumab emtansine (Kadcyla; T-DM1), which was the first ADC approved for treating HER2+ metastatic BC. T-DM1 demonstrated significant improvements in disease-free survival (DFS) and overall survival (OS) in pivotal trials such as KATHERINE (NCT01772472) and DESTINY-Breast03 (NCT03529110)^[15]^. Further trials, such as TH3RESA (NCT01419197) and MARIANE (NCT01120184), confirmed the effectiveness of T-DM1 in heavily pretreated patients^[16]^.

## GENERAL ADC RESISTANCE MECHANISMS

**Figure 1:**
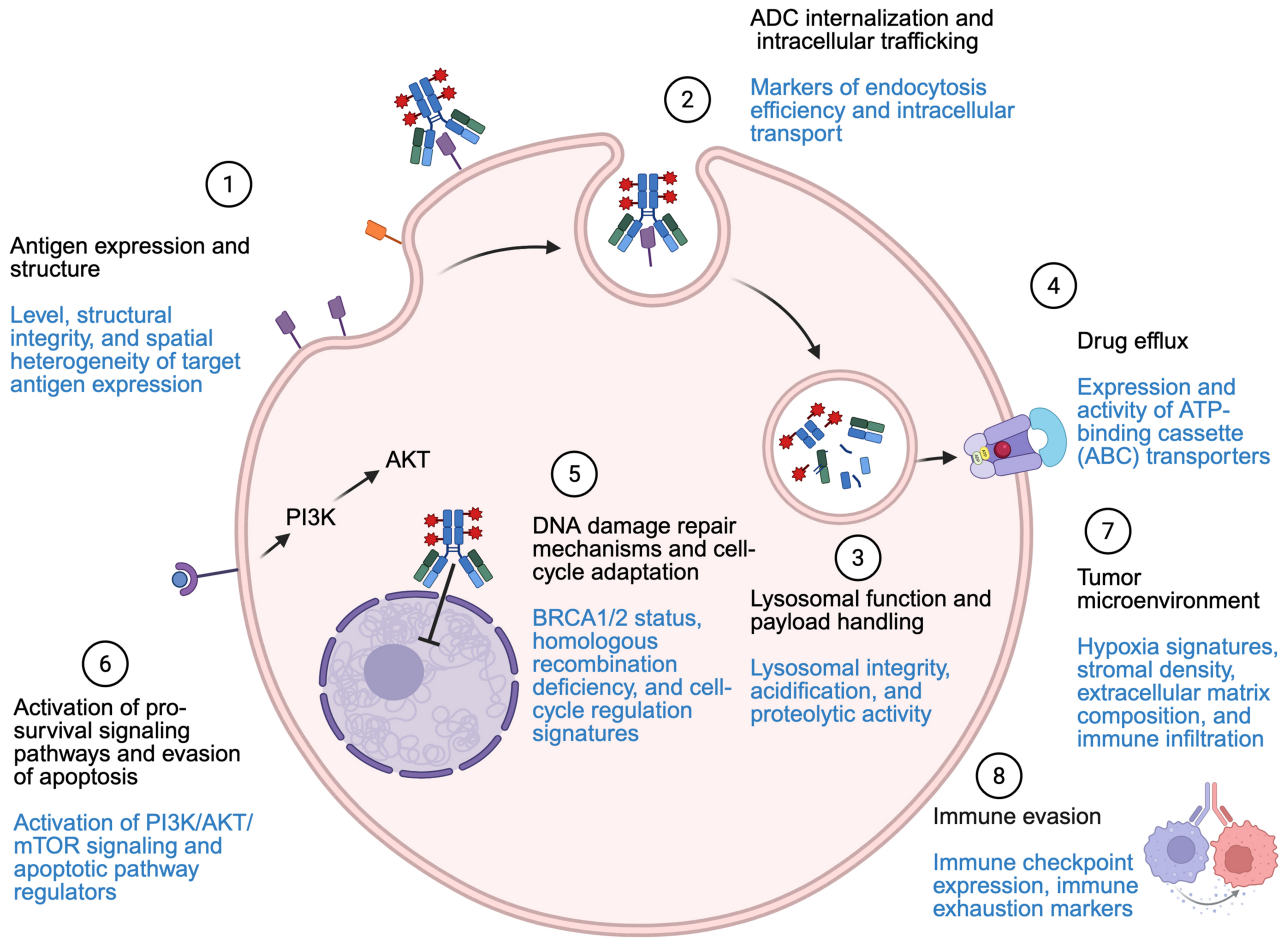
Resistance mechanisms and proposed biomarkers (highlighted in blue). Created in BioRender. Marina, A. (2026) https://BioRender.com/hdkg3fu. ADC: Antibody-drug conjugate; ATP: adenosine triphosphate; BRCA1/2: BReast CAncer gene (BRCA)1/2; PI3K: phosphoinositide 3-kinase; AKT: protein kinase B; mTOR: mammalian target of rapamycin.

Because of the complexity and diversity of both target antigens and cytotoxic payloads, the mechanisms by which tumors develop resistance to ADCs are varied and often require tailored investigation for each specific ADC^[17]^. The success of ADCs relies on the properties of their three main components: the antibody, the cytotoxic payload, and the linker [Figure 1].

### Antigen-related resistance

One major contributor to resistance is the reduction or mutation of the targeted receptor detected by the antibody, which diminishes the delivery of the cytotoxic payload. To counteract this, one approach is to increase the expression of the target antigen^[18-21]^. Although changing the target has led to some improvements in progression-free survival (PFS), these results have not reached statistical significance, highlighting the need for further research.

Beyond quantitative changes, qualitative modifications of target antigens also contribute to resistance. Structural alterations, including truncation of extracellular domains, alternative splicing, point mutations, or epitope masking, can impair antibody recognition, thereby promoting therapeutic escape^[22]^.

To improve efficacy, soluble ligands have been developed that allow the release and diffusion of the payload across cell membranes, enabling the drug to affect neighboring cells, even those lacking the target antigen^[20,23]^. Furthermore, novel ADC designs are being explored, including bispecific antibodies, double-charge ADCs, and smaller molecular constructs. Bispecific antibodies can bind to different sites on the same antigen or to two separate antigens. ADCs with dual payloads use two distinct, synergistic cytotoxic agents to reduce the likelihood of resistance.

Changes in antigen expression or structure may explain why subsequent ADCs lose efficacy. This underscores the importance of optimizing ADC sequencing and incorporating biomarker-based strategies to improve patient outcomes and overcome resistance^[23,24]^.

### Altered intracellular trafficking and lysosomal dysfunction

A challenge for ADCs is their large molecular size, which limits their ability to penetrate tumors efficiently, meaning only a small proportion of the drug reaches tumor cells. One solution is to attach the payload to an antibody fragment, which decreases the overall size of the ADC and enhances tumor penetration and drug delivery^[21,23]^. Also, disruptions in vesicular transport or lysosomal function can significantly impair ADC efficacy. Defective lysosomal acidification or reduced proteolytic enzyme activity may prevent efficient linker cleavage, resulting in incomplete payload release and diminished cytotoxicity^[25]^.

### Alterations in payload targets and cytotoxic activity

Mutations in enzymes targeted by ADC payloads, such as topoisomerase I, can reduce drug binding or activity^[26]^. In addition, dysregulation of cell cycle regulators and mitotic kinases has been implicated in resistance to ADC-induced cytotoxicity^[27]^. Tumor cells may bypass cell cycle arrest or DNA damage responses triggered by the payload, allowing continued proliferation^[28]^.

### Resistance to apoptosis and activation of survival pathways

Even when payloads successfully reach their intracellular targets, tumor cells may evade cell death through dysregulation of apoptotic signaling^[29]^. Loss of pro-apoptotic proteins or overexpression of anti-apoptotic factors can blunt ADC-induced apoptosis. Activation of survival pathways, including the phosphatidylinositol 3-kinase (PI3K)/protein kinase B (AKT)/mammalian target of rapamycin (mTOR) signaling cascade, further promotes resistance by enhancing cell survival and reducing sensitivity to cytotoxic stress^[30]^. Notably, alternative forms of cell death have also been implicated in ADC resistance. Emerging evidence suggests that modulation of ferroptosis and oxidative stress pathways may influence sensitivity to certain ADCs.

### Drug efflux

Overexpression of drug efflux transporters, particularly ABC transporters, can actively expel cytotoxic agents from tumor cells. Enhanced efflux reduces intracellular drug concentration and limits the effectiveness of ADC payloads. Preclinical studies have demonstrated that pharmacological inhibition of efflux transporters can partially restore sensitivity.

### Influence of the tumor microenvironment

The tumor microenvironment plays a critical role in modulating ADC efficacy and resistance. Cancer-associated fibroblasts can restrict ADC penetration through extracellular matrix remodeling and downregulation of target antigens. Tumor-associated macrophages may reduce ADC availability through Fc receptor–mediated uptake and degradation while promoting an immunosuppressive milieu. Conversely, ADC-induced immunogenic cell death can enhance antitumor immunity by activating CD8^+^ T cells and natural killer cells; however, immune exhaustion and checkpoint activation often limit this effect^[31]^. Combination strategies integrating ADCs with immunomodulatory therapies are therefore being actively explored^[32]^.

Therefore, ongoing research focused on addressing mechanisms of resistance, optimizing linker and payload technologies, and broadening their clinical applicability across a wider range of tumor types and patient populations^[16,33]^.

## CLINICAL MANAGEMENT OF TNBC

TNBC represents a particularly aggressive form of BC, comprising around 15% of all diagnosed cases. It is defined by the absence of ER, PR, and HER2, which makes it unresponsive to many of the targeted therapies available for other BC subtypes^[2]^.

TNBC is known for its poor clinical outcomes due to its invasive behavior and resistance to many conventional therapies^[34]^. The standard approach for high-risk TNBC typically involves anthracycline-based chemotherapy regimens that combine drugs such as doxorubicin and cyclophosphamide with taxanes such as paclitaxel or docetaxel. In patients with stage II or III disease, neoadjuvant chemotherapy is preferred to shrink tumors before surgery, reduce the extent of surgical intervention, and allow for treatment adjustments based on the pathological response. Achieving a pathological complete response is a key indicator of favorable prognosis, while the presence of residual disease after neoadjuvant therapy is linked to an increased risk of recurrence. In response to this challenge, ongoing research has explored additional therapies for patients who do not achieve pathological complete response. The Capecitabine for Residual Cancer as Adjuvant Therapy (CREATE-X) trial demonstrated improved outcomes when capecitabine was added to the treatment regimen of TNBC patients with residual invasive disease after standard neoadjuvant chemotherapy^[35]^. Furthermore, the use of poly(adenosine diphosphate-ribose) polymerase (PARP) inhibitors, such as olaparib and talazoparib, has shown promise in patients with BReast CAncer gene (*BRCA*)*1/2* mutations or metastatic and high-risk TNBC^[36,37]^. Immunotherapy has also emerged as a potential option^[38]^; agents such as pembrolizumab and atezolizumab have been investigated in combination with chemotherapy for patients with advanced or treatment-naïve disease^[39]^.

## OVARIAN CANCER: CLINICAL EVIDENCE AND FUTURE PERSPECTIVES

Ovarian carcinoma is among the most aggressive and lethal gynecological malignancies, and most cases are diagnosed in advanced stages, thereby complicating clinical management and significantly reducing patient survival. The current standard of care for advanced ovarian carcinoma involves a combination of cytoreductive surgery and systemic chemotherapy^[40]^.

Therapeutic response is influenced by various genetic and epigenetic factors, including *BRCA1/2* mutations, Cyclin E1 (*CCNE1*) amplification, and the tumor’s immunological profile. Significant advances have been made in the development of targeted therapies, particularly PARP inhibitors such as olaparib, niraparib, and rucaparib, which have demonstrated efficacy in patients harboring *BRCA* mutations. Moreover, the exploration of combination regimens with immunotherapy is ongoing^[40]^. Nevertheless, surgical intervention continues to represent a cornerstone of advanced ovarian carcinoma treatment. The major clinical challenge lies in personalizing therapy to enhance survival outcomes while minimizing toxicity. These efforts have catalyzed the investigation of novel therapeutic strategies, among which ADCs have emerged as particularly promising^[4]^. The therapeutic potential of ADCs in gynecologic malignancies has been exemplified by the approval of tisotumab vedotin for recurrent or metastatic cervical cancer.

## TROP2

Among the various antigens under investigation for the development of new ADCs, the TROP2 has attracted considerable attention as a therapeutic target. TROP2 is a 36 kDa transmembrane glycoprotein belonging to the EpCAM family and encoded by the tumor associated calcium signal transducer 2 (*TACSTD2*) gene located on the short arm of chromosome 1 (1p32.1). It functions as a cell surface receptor mediating intracellular calcium signaling. Initially identified as a marker on trophoblast cells of the placenta and fetal tissues. TROP2 overexpression has been reported in diverse solid tumors including colorectal, renal, lung, breast (particularly TNBC subtype), and ovarian carcinoma as well as in rare aggressive malignancies such as anaplastic thyroid carcinoma, salivary duct carcinoma or neuroendocrine prostate cancers. TROP2 overexpression results from both transcriptional and posttranscriptional deregulations, and is associated with less differentiated tumor cells, metastasis, higher tumor grade, and poorer prognosis. TROP2 contributes to tumor progression by engaging key signaling pathways associated with malignancy such as AP-1, which regulates gene expression involved in carcinogenesis, proliferation, migration, metastasis, angiogenesis, apoptosis, and epithelial-to-mesenchymal transition.

Because of its high expression on the surface of various tumor types and limited presence in normal tissues, TROP2 is an attractive target for therapy. Reducing TROP2 levels impairs tumor cell proliferation, emphasizing its potential as a prognostic biomarker for high-risk patients and as a therapeutic target for advanced cancers.

## ADCS TARGETING TROP2

Although TROP2 overexpression is observed across all BC subtypes, recent research by Aslan *et al*. has demonstrated that its expression is most pronounced in TNBC (85%) when compared to HR+ and HER2+ subtypes^[41]^. Published studies indicate that TROP2 is also overexpressed in up to 96% of endometrial cancers and in approximately 65% of uterine serous carcinomas. Furthermore, high levels of TROP2 expression have been observed in ovarian carcinoma. Bignotti *et al*. found that TROP2 staining was positive in 92% of ovarian carcinoma tumors, compared to only 15% in normal ovarian surface epithelium samples. Moreover, TROP2 overexpression was associated with poorer OS and PFS in ovarian carcinoma patients, supporting its potential as a therapeutic target^[42]^. In response to this finding, ADCs targeting TROP2, such as SG, Dato-DXd, and others, have emerged in recent years as promising therapeutic strategies for the treatment of metastatic BC and other cancers [Table 1].

**Table 1:** TROP2-targeting ADCs as monotherapy in clinical trials

**Clinical assay name**	**Drug**	**Target tumor**	**Patient characteristics**	**Phase**	**Patient number**	**Status**	**Key results**	**Ref.**
IMMU-132-01 (NCT01631552)	SG *vs.* TPC	MULTI	Solid epithelial tumors, including gynecologic malignancies and mTNBC	I/II	515	Completed	ORR: 33.3%; DoR: 7.7 m; PFS: 5.6 m; OS: 13 m	[43]
ASCENT (NCT02574455)	SG *vs.* TPC	BC	TNBC advanced with two or more previous lines of QT	III	529	Completed	ORR: 35%; PFS: 5.6 m; OS: 12.1 m	[44]
TROPiCS-02 (NCT03901339)	SG *vs.* TPC	BC	Endocrine-resistant HR+/HER2- mBC who have failed at least two prior QT regimens	III	543	Completed	ORR: 21%; CBR: 34%; PFS: 5.5 m; OS: 14.5 m	[45]
SASCIA (NCT04595565)	SG *vs.* platinum-based QT	BC	TNBC or ER/PR+ BC and residual disease after primary therapy	III	1,332	Active, not recruiting	NA	[47]
ASCENT-03 (NCT05382299)	SG *vs.* TPC	BC	ABC, TNBC who received anti-PD-L1 in the neo/adjuvant setting	III	623	Recruiting. Estimated primary completion: June 2028	NA	[46]
NCT06028932	SG	OV	Platinum-resistant OV	II	20	Recruiting	NA	[51]
NCT04251416	SG	ENDO	Recurrent endometrial carcinoma that is refractory to platinum or has progressed after platinum-based QT treatment	II	50	Recruiting	NA	-
TROPiCS-03 (NCT03964727)	SG	MULTI	Patients had previous treatment with platinum-based QT and immunotherapy	II	227	Active, not recruiting	NA	[49]
GOG-3104/ENGOT-en26 (NCT06486441)	SG *vs.* TPC	ENDO	Recurrent ENDO previously treated with platinum-based QT and anti-PD-1/anti-PD-L1 therapy	III	640	Recruiting. Estimated study completion: June 2029	NA	-
EVOKE-01 (NCT05089734)	SG *vs.* docetaxel	LUNG	NSCLC	III	603	Completed	ORR: 13.7%; DoR: 6.7 m; PFS: 4.1 m; OS: 11.1 m	[48]
NCT05101096 (ASCENT-J02)	SG	MULTI	Advanced solid tumors/mTNBC, HR+ HER- mBC, mUC. Japanese participants	I/II	135	Active, not recruiting	NA	-
TROPION-Breast01 (NCT05104866)	Dato-DXd *vs.* TPC (QT)	BC	ER/PR+ ABC previously treated with 1-2 lines of QT	III	732	Completed	ORR: 36.4%; DoR: 6.7 m; PFS: 6.9 m	[56]
TROPION-Breast02 (NCT05374512)	Dato-DXd *vs.* TPC	BC	Locally recurrent inoperable or mTNBC are not candidates for PD-1/PD-L1 therapy	III	644	Active, not recruiting	NA	[57]
TROPION-PanTumor01 (NCT03401385)	Dato-DXd	MULTI	Advanced heavily pretreated solid tumors, including advanced TNBC	I	890	Completed	ORR: 26.8%/31.8%^*^; DCR: 80%; PFS: 4.3 m; CBR: 34%; OS: 12.9 m	[52]
TROPION-PanTumor02 (NCT05460273)	Dato-DXd	MULTI	Advanced or metastatic solid tumors, including TNBC. Chinese patients	I/II	119	Completed	ORR: 33.8%; DoR: 5.8 m; PFS: 5.3 m; OS: 13.5 m^*^	-
TROPION-PanTumor03 (NCT05489211)	Dato-DXd monotherapy and Dato-DXd + anticancer agents	MULTI	Advanced/metastatic solid tumors (endometrial and ovarian cohorts)	II	582	Recruiting	NA	[55]
MK-2870-020/TroFuse-020/GOG-3101/ ENGOT-cx20 (NCT06459180)	Sac-TMT *vs.* TPC	CERVI	Recurrent CERVI previously treated with systemic platinum doublet QT (with or without bevacizumab) and anti-PD-1/anti-PD-L1 therapy	III	686	Recruiting. Estimated primary completion: October 2028	NA	-
ENGOT-en23/GOG-3095/MK-2870-005 (NCT06132958)	Sac-TMT *vs.* QT	ENDO	ENDO patients who had received prior platinum-based QT and/or anti-PD-L1 immunotherapy	III	710	Recruiting. Estimated primary completion: October 2028	NA	[59]
NCT04152499	Sac-TMT	MULTI	Locally advancer tumors refractory or metastatic (ovarian, TNBC, NCLS, gastric adenocarcinoma)	I/II	1,410	Completed	ORR (TNBC): 34.8%-38.9%; ORR (HR+/HER2- BC): 31.7%	[60]

^*^TNBC patient data. TROP2: Trophoblast cell-surface antigen 2; ADCs: antibody-drug conjugates; SG: sacituzumab govitecan; TPC: treatment of physician’s choice; MULTI: multiple cancers; mTNBC: metastatic triple-negative breast cancer; ORR: objective response rate; DoR: duration of response; PFS: progression-free survival; OS: overall survival; BC: breast cancer; QT: chemotherapy; HR+: hormone receptor-positive; HER2-: human epidermal growth factor receptor 2-negative; mBC: metastatic breast cancer; CBR: clinical benefit rate; ER: estrogen; PR+: progesterone receptor-positive; NA: not available; ABC: adenosine triphosphate-binding cassette; PD-L1: programmed death ligand 1; OV: ovarian cancer; ENDO: endometrial cancer; PD-1: programmed cell death 1; LUNG: lung cancer; NSCLC: non-small cell lung cancer; mUC: metastatic urothelial cancer; Dato-DXd: datopotamab deruxtecan; DCR: disease control rate; CERVI: cervical cancer; NCLS: non-small cell lung cancer.

### IMMU-132/SG: clinical development

SG is an ADC that links a topoisomerase I inhibitor (SN-38) to an antibody targeting TROP2. This design enables selective drug delivery to TROP2-expressing tumor cells while minimizing systemic exposure.

SG was first investigated in metastatic TNBC in the phase I/II IMMU-132-01 trial (NCT01631552) and later in the phase III ASCENT study (NCT02574455). In IMMU-132-01, heavily pretreated patients (median of three prior chemotherapy regimens) achieved an objective response rate (ORR) of 33%, with median PFS of 5.5 months and median OS of 13 months, supporting the FDA’s accelerated approval. Grade 3-4 adverse events, primarily anemia and neutropenia, occurred in about 10% of patients, indicating a manageable safety profile^[43]^.

In the ASCENT phase III trial, SG was compared with the treatment of physician’s choice (TPC) (eribulin, vinorelbine, or gemcitabine) in patients with advanced TNBC previously exposed to at least two chemotherapy regimens. SG significantly prolonged both PFS and OS, particularly in patients without brain metastases, and confirmed its role as a preferred option in this population^[44]^.

In the TROPiCS-02 trial (NCT03901339), SG was evaluated against TPC (capecitabine, vinorelbine, gemcitabine, or eribulin) in ER/PR+ advanced BC previously treated with cyclin-dependent kinase (CDK)4/6 inhibitors and at least two lines of chemotherapy. SG demonstrated a meaningful clinical benefit but showed heterogeneous responses across patients, and grade 3 adverse events, mainly neutropenia, diarrhea, and anemia, occurred in 74% of patients receiving SG *vs*. 60% with standard therapy^[45]^.

The ASCENT-03 trial (NCT05382299) is currently evaluating SG as first-line therapy in patients with advanced BC or TNBC, including both programmed death ligand 1 (PD-L1)+ and PD-L1- subgroups. This trial will clarify whether earlier use of SG can further improve outcomes in advanced disease^[46]^.

Beyond the metastatic setting, ADCs are being investigated in early-stage BC to reduce recurrence. The phase III SASCIA trial (NCT04595565) randomizes patients with residual TNBC after initial therapy to receive SG or platinum-based chemotherapy, providing an opportunity to assess the impact of SG in high-risk early disease^[47]^.

Additional clinical studies are testing SG as monotherapy in other solid tumors. For example, the phase III EVOKE-01 (NCT05089734) includes patients with non-small cell lung cancer (NSCLC)^[48]^. Encouraging results have been reported for SG in TNBC and urothelial carcinoma, with ongoing trials extending its use to gynecologic malignancies such as ovarian and endometrial cancers. The phase II TROPiCS-03 trial (NCT03964727) includes patients with metastatic solid tumors previously treated with platinum-based chemotherapy and immunotherapy, with a dedicated cohort for endometrial carcinoma. This trial remains ongoing, although it is no longer recruiting participants, and is expected to further define the role of SG in heavily pretreated solid tumors^[49]^.

In ovarian carcinoma, preclinical models have shown SG can effectively induce tumor regression in TROP2-expressing tumors, and early-phase trials confirmed a manageable safety profile and promising efficacy, particularly in patients with high TROP2 expression^[50]^. The ongoing phase II NCT06028932 trial in platinum-resistant ovarian carcinoma is anticipated to refine the positioning of SG in this challenging setting^[51]^.

### Dato-DXd: clinical development

Dato-DXd is a next-generation anti-TROP2 ADC, composed of a humanized IgG1 monoclonal antibody targeting TROP2, linked via a tetrapeptide-based cleavable linker to DXd, a highly potent topoisomerase I inhibitor derived from exatecan. The linker is engineered to remain stable in systemic circulation, ensuring that the cytotoxic payload is predominantly released inside tumor cells following internalization^[52]^.

Pharmacologically, Dato-DXd benefits from a drug-to-antibody ratio of 4:1, balancing potency and tolerability. Its DXd payload is substantially more potent than SN-38 (used in SG), and the ADC exhibits a long half-life with primarily biliary-fecal elimination. These properties facilitate convenient triweekly dosing and reduce premature systemic exposure^[53]^.

Clinical evaluation began with the TROPION-PanTumor01 phase I study in heavily pretreated metastatic or advanced TNBC. The overall ORR was 32%, increasing to 44% among patients naïve to prior topoisomerase I inhibitor ADCs. The safety profile was manageable, with grade 3 or higher adverse events occurring in ~50% of patients, most commonly stomatitis, nausea, vomiting, and fatigue. Importantly, no treatment-related deaths were observed^[52,54]^.

Subsequent trials, including TROPION-PanTumor02 (in Chinese patients) and TROPION-PanTumor03, expanded evaluation to endometrial and ovarian carcinomas. In endometrial cancer, the ORR was 27.5%, with a disease control rate (DCR) of 85%. In ovarian cancer, the ORR was 42.9% and the DCR 91.4%, with both cohorts showing moderate median duration of response and PFS^[55]^.

Phase III studies are ongoing to confirm efficacy and safety in BC. TROPION-Breast01 compared Dato-DXd with standard chemotherapy in metastatic TNBC patients with one or two prior regimens, showing improved median PFS (6.9 *vs*. 4.9 months) and higher ORR (36.4% *vs*. 22.9%) in favor of Dato-DXd^[56]^.

TROPION-Breast02 is evaluating Dato-DXd in locally recurrent or metastatic TNBC in patients ineligible for programmed cell death 1 (PD-1)/PD-L1 therapy. These trials will help refine patient selection and may guide integration of Dato-DXd into treatment algorithms for advanced TNBC^[57]^.

Overall, Dato-DXd shows promising antitumor activity across multiple solid tumors. Ongoing research aims to optimize its use through biomarker-driven strategies and rational combinations to enhance the durability of clinical benefit.

### Sacituzumab tirumotecan: mechanistic advancements

Sacituzumab tirumotecan (Sac-TMT, also known as SKB264) is a novel TROP2-directed ADC designed to overcome some of the pharmacological and resistance-related limitations observed with earlier TROP2-targeted ADCs such as SG. Similar to SG, Sac-TMT employs the same monoclonal antibody targeting TROP2 but incorporates a distinct cytotoxic payload, KL610023 (T030), a belotecan-derived topoisomerase I inhibitor, linked via disulfide bonds. This structural modification enhances plasma stability, tumor-specific accumulation, and bystander cytotoxicity, potentially improving payload delivery in tumors with heterogeneous or low TROP2 expression^[58]^.

Preclinical studies have demonstrated potent antitumor activity in TROP2+ patient-derived xenograft models. This may result from improved payload release dynamics and enhanced membrane permeability of the KL610023 moiety that facilitates killing of neighboring tumor cells.

Clinically, Sac-TMT is being explored across multiple gynecologic malignancies, where resistance to platinum-based chemotherapy and immune checkpoint blockade remains a major challenge. The phase III ENGOT-en23/GOG-3095/MK-2870-005 trial is comparing Sac-TMT with chemotherapy in patients with endometrial carcinoma or carcinosarcoma previously treated with platinum agents and/or anti–PD-L1 immunotherapy^[59]^. Another phase III study (MK-2870-020/TroFuse-020/GOG-3101/ENGOT-cx20) is evaluating Sac-TMT against the TPC in recurrent cervical cancer following prior platinum doublet and PD-1/PD-L1 therapy. These trials aim to define the role of Sac-TMT as a new therapeutic option in heavily pretreated gynecologic cancers.

In the phase I/II NCT04152499 trial involving patients with unresectable, locally advanced, or metastatic solid tumors, including TNBC and HR+/HER2- BC, Sac-TMT demonstrated ORR of 34.8%-38.9% in TNBC and 31.7% in HR+/HER2- disease. The safety profile was manageable, with mainly grade 1-2 nausea, alopecia, anemia, stomatitis, and vomiting. Grade 3-4 adverse events occurred in 57% of patients, and no grade 5 toxicities were observed.

Overall, the clinical activity of Sac-TMT in heavily pretreated populations supports its development as a next-generation TROP2-directed ADC in breast and gynecologic malignancies. Ongoing phase III studies will further clarify its comparative efficacy and long-term safety relative to standard therapies^[60]^.

## ADCS TARGETING TROP2 IN COMBINATION THERAPIES

Combination strategies are increasingly being explored to enhance the therapeutic efficacy of TROP2-targeting ADCs and overcome mechanisms of acquired or intrinsic resistance. To this end, a range of rational combination strategies is under evaluation, pairing ADCs with immune checkpoint inhibitors, anti-angiogenic therapies, PARP inhibitors, or other targeted agents to achieve synergistic antitumor effects and re-sensitize resistant tumors [Table 2]. In parallel, the implementation of companion diagnostic tools to assess antigen expression has become essential for identifying patients most likely to benefit from ADC-based treatments and for supporting precision-guided therapeutic decision making^[7]^.

**Table 2:** TROP2-targeting ADCs in combination therapy in clinical trials

**ADC drug**	**Clinical assay name**	**Drug combination**	**Targets**	**Tumor type**	**Phase**	**Patient number**	**Status**	**Key results**	**Ref.**
SG	NCT03992131	SG + PARP inhibitors (rucaparib, lucitanib)	TROP2 + PARP	TNBC, OV, UC, solid tumor	Ib/II	25	Completed	Not analysed	[61]
SG	NCT04039230	SG + talazoparib	TROP2 + PARP	mTNBC	Ib/II	75	Recruiting	NA	[62]
SG	NCT05113966	SG + CDK4/6 inhibitors (trilaciclib)	TROP2 + CDK4/6	mTNBC	II	30	Completed	ORR: 23.3%; PFS: 4.1 m; OS: 15.9 m; CBR: 46.7%	[63]
SG	NCT04958785	SG + CD47 antibody (magrolimab) *vs.* magrolimab + paclitaxel or Nab-paclitaxel	TROP2 + CD47	Non-surgically removable locally advanced or mTNBC	II	92	Completed	NA	[64]
SG	NCT05008510	SG *vs.* SG + sabizabulin	TROP2 + tubulin	mTNBC	II	0	Withdrawn		-
SG	NCT02161679	SG *vs.* SG + carboplatin (QT)	TROP2	TNBC	II	0	Withdrawn		-
SG	NCT04927884	SG + cyclophosphamide (QT)	TROP2	TNBC with at least two prior treatments for metastatic disease	Ib/II	3	Completed	Low enrollment	-
SG	NEOSTAR (NCT04230109)	SG *vs.* SG + pembrolizumab	TROP2 + PD-1	Neoadjuvant in localized TNBC	II	260	Recruiting	pCR rate 30%, ORR 64%	[65]
SG	Saci-IO TNBC (NCT04468061)	SG *vs.* SG + pembrolizumab	TROP2 + PD-1	PD-L1-negative mTNBC	II	110	Recruiting	NA	-
SG	ASCENT-04 (NCT05382286)	SG + pembrolizumab *vs.* pembrolizumab + TPC	TROP2 + PD-1	Untreated locally ABC/TNBC with PD-L1 expression	III	443	Active, not recruiting	NA	[66]
SG	Morpheus-TNBC (NCT03424005)	SG + atezolizumab *vs.* SGN-LIV1A + atezolizumab	TROP2 + PD-L1	Inoperable ABC or mTNBC	Ib/II	792	Recruiting. Estimated primary completion: May 2028	NA	[69]
SG	NCT04434040	SG + atezolizumab	TROP2 + PD-L1	TNBC	II	40	Active, not recruiting	NA	-
SG	InCITe (NCT03971409)	SG + avelumab *vs.* avelumab + liposomal doxorubicin	TROP2 + PD-L1	mTNBC, stage IV or unresectable	II	150	Recruiting	NA	-
SG	ASCENT-05/AFT-65 OptimICE-RD (NCT05633654)	SG + pembrolizumab *vs.* TPC (pembrolizumab or pembrolizumab + capecitabine)	TROP2 + PD-1	mTNBC with two or more standard QT treatments	III	1,514	Recruiting. Estimated primary completion: June 2027	NA	[67]
SG	Saci-IO HR+ (NCT04448886)	SG *vs.* SG + pembrolizumab	TROP2 + PD-1	HR+/HER2- mBC	II	110	Completed	ORR:28.8 %; PFS: 8.36 m; CBR: 50%	[68]
SG	NCT05675579	Neoaduvant SG + pembrolizumab	TROP2 + PD-1	Immunohemotherapy-resistant early-stage TNBC	II	27	Recruiting. Estimated primary completion: December 2026	NA	-
SG	ADAPT-TN-III (NCT06081244)	SG *vs.* SG + pembrolizumab	TROP2 + PD-1	Low-risk early TNBC	II	348	Recruiting. Estimated primary completion: September 2029	NA	-
SG	NCT06878625	SG + toripalimab *vs.* SG + toripalimab + bevacizumab	TROP2 + PD-1 + angiogenesis	mTNBC	II	138	Recruiting. Estimated primary completion: June 2027	NA	-
SG	NCT05143229	SG + alpelisib	TROP2 + PI3K alpha	Metastatatic or locally recurrent HER2-negative BC	I	18	Recruiting	NA	-
SG	NCT06040970	SG + cisplatin	TROP2	Platinum sensitive recurrent OV and ENDO	I/II	54	Recruiting. Estimated primary completion: September 2026	NA	-
SG	NCT05006794	SG + GS-9716 *vs.* GS-9716 + docetaxel	TROP2 + MCL1	Solid malignancies	Ia/b	145	Active, not recruiting. Estimated primary completion: March 2029	NA	-
SG	NCT05867251	AVZO-021 *vs.* AVZO-021 + SG	TROP2 + CDK2	Advanced solid tumors (OV, TNBC, ENDO, fallopian tube cancer, HR+/HER2- BC)	I/II	430	Recruiting. Estimated primary completion: January 2028	NA	[72]
Dato-DXd	BEGONIA (NCT03742102)	Dato-DXd + durvalumab *vs.* Dato-DXd + durvalumab + paclitaxel	TROP2 + PD-L1	mTNBC	Ib/II	243	Active, not recruiting. Estimated completion: February 2027	NA	[73]
Dato-DXd	TROPION-Breast03 (NCT05629585)	Dato-DXd *vs.* Dato-DXd + durvalumab or TPC	TROP2 + PD-L1	I-III TNBC with residual disease following neoadjuvant therapy with anthracycline and taxane	III	1,174	Active, not recruiting. Estimated primary completion: September 2027	NA	[74]
Dato-DXd	TROPION-Breast05 (NCT06103864)	Dato-DXd/Dato-DXd + durvalumab *vs.* TPC (QT + pembrolizumab)	TROP2 + PD-L1/PD-1	mTNBC	III	625	Recruiting. Estimated primary completion: December 2026	NA	[75]
Dato-DXd	TROPION-Breast04 (NCT06112379)	Dato-DXd + durvalumab *vs.* pembrolizumab + QT	TROP2 + PD-L1/PD-1	mTNBC	III	1,902	Recruiting. Estimated primary completion: November 2028	NA	[76]
Dato-DXd	TRADE-DXd (NCT06533826)	Dato-DXd followed T-DXd *vs.* T-DXd followed Dato-DXd (COMBI sequence)	TROP2 + HER2	ADC-refractory BC which will include patients with HER2-low ABC	II	357	Recruiting. Estimated primary completion: January 2028	NA	-
Dato-DXd	DESTINITY-Ovarian01 (NCT06819007)	Dato-DXd + bevacizumab *vs.* bevacizumab	TROP2 + angiogenesis	First-line maintenance of HER2-expressing advanced high-grade OV	III	582	Recruiting. Estimated primary completion: November 2028	NA	-
Dato-DXd	NCT05417594	AZD9574 *vs.* AZD9574 + anticancer agents (Dato-DXd)	TROP2 + PARP1	Advanced solid tumors (ovarian, breast, pancreatic, prostate cancers)	I/II	695	Recruiting. Estimated primary completion: February 2027	NA	-
Dato-DXd	NCT04644068	AZD5305 *vs.* AZD5305 + anticancer agents (Dato-DXd)	TROP2 + PARP1	Advanced solid tumors (ovarian, breast, pancreatic, prostate, colon, gastric, bladder, cervical, endometrial cancers)	I/II	702	Recruiting. Estimated primary completion: June 2027	NA	-
R-DXd	MK-5909-003 (NCT06843447)	R-DXd *vs.* R-DXd + anticancer agents (carboplatin, paclitaxel or bevacizumab)	Angiogenesis	High-grade serous OV, primary peritoneal or fallopian tube cancer who had relapse after prior platinum-based QT	Ib/II	280	Recruiting. Estimated primary completion: March 2029	NA	-
Sac-TMT	NCT05642780	Sac-TMT + pembrolizumab	TROP2 + PD-1	Previously treated CERVI with recurrent disease	II	240	Recruiting. Estimated primary completion: November 2026	NA	-
Sac-TMT	MK-2870-022/TroFuse-022/GOG-3103/ ENGOT-ov84 (NCT06824467)	Sac-TMT/Sac-TMT + bevacizumab *vs.* TPC	TROP2 + angiogenesis	Platinum-sensitive recurrent OV	III	770	Recruiting. Estimated primary completion: April 2029	NA	-

TROP2: Trophoblast cell-surface antigen 2; ADCs: antibody-drug conjugates; SG: sacituzumab govitecan; PARP: poly(adenosine diphosphate-ribose) polymerase; TNBC: triple-negative breast cancer; OV: ovarian cancer; UC: urothelial cancer; mTNBC: metastatic triple-negative breast cancer; NA: not available; ORR: objective response rate; PFS: progression-free survival; OS: overall survival; CBR: clinical benefit rate; QT: chemotherapy; PD-1: programmed cell death 1; pCR: pathological complete response; PD-L1: programmed death ligand 1; TPC: treatment of physician’s choice; ABC: adenosine triphosphate-binding cassette; HR+: hormone receptor-positive; HER2-: human epidermal growth factor receptor 2-negative; mBC: metastatic breast cancer; PI3K: phosphoinositide 3-kinase; BC: breast cancer; ENDO: endometrial cancer; MCL1: myeloid cell leukemia 1; Dato-DXd: datopotamab deruxtecan; R-DXd: raludotatug deruxtecan; Sac-TMT: sacituzumab tirumotecan; CERVI: cervical cancer.

### SG combinatory therapies

PARP inhibitor combinations (e.g., rucaparib, lucitanib, talazoparib; NCT03992131, NCT04039230) aim to exploit synthetic lethality by inhibiting DNA repair in tumor cells already stressed by SG-induced DNA damage^[61,62]^.

Similarly, combinations of SG with CDK4/6 inhibitors such as trilaciclib (NCT04958785) are designed to disrupt cell cycle–dependent repair mechanisms and enhance ADC-induced cytotoxicity^[63]^. Pairing SG with CD47 blockade (magrolimab; NCT04958785) may enhance antitumor activity by promoting phagocytic clearance of ADC-treated tumor cells and modulating innate immune responses^[64]^.

Given the importance of immune modulation, multiple trials are evaluating SG in combination with immune checkpoint inhibitors to amplify adaptive antitumor immunity. For example, the NEOSTAR trial (NCT04230109) and NCT04468061 are investigating SG plus pembrolizumab in both localized and metastatic TNBC, aiming to increase pathologic response and long-term tumor control^[65]^.

The phase III ASCENT-04 study (NCT05382286) is comparing SG plus pembrolizumab with standard pembrolizumab-based therapy in PD-L1+ TNBC, testing whether immune checkpoint blockade can potentiate ADC activity and improve survival outcomes^[66]^. Additional studies include combinations with atezolizumab (NCT04434040), avelumab (InCITe; NCT03971409), pembrolizumab (ASCENT-05; NCT05633654)^[67]^ and toripalimab ± bevacizumab (NCT06878625), extending the evaluation of SG-based combinations across the TNBC spectrum.

NCT05675579 and ADAPT-TN-III (NCT06081244) are assessing SG plus pembrolizumab in early or resistant TNBC to determine whether combination therapy can improve cure rates and delay relapse.

The Morpheus-TNBC trial (NCT03424005) compares SG with SGN-LIV1A, another ADC targeting LIV-1 (SLC39A6), both combined with atezolizumab, reflecting broader efforts to diversify antigenic targets in TNBC.

SGN-LIV1A is a humanized monoclonal antibody directed against the zinc transporter LIV-1 (SLC39A6) and conjugated via a protease-cleavable linker to monomethyl auristatin E, a potent microtubule-disrupting agent. LIV-1 is upregulated in TNBC and remains expressed after hormonal therapy in ER+ BC, suggesting a potential role where TROP2-targeted therapies are insufficient. This strategy exemplifies how alternative ADCs may complement or replace TROP2-directed agents in specific clinical contexts^[68]^.

Beyond immune combinations, SG is also being tested with PI3K inhibition (alpelisib; NCT05143229) to evaluate pathway-targeted combinations in BC^[69]^ and with cisplatin (NCT06040970) in platinum-sensitive ovarian and endometrial cancers to exploit DNA damage synergy^[70]^. The addition of the MCL-1 inhibitor GS-9716 (NCT05006794) is being investigated to enhance apoptosis induction in SG-treated tumors.

The phase I/II study NCT05867251 is evaluating SG with AVZO-021, a selective CDK2 inhibitor, across multiple solid tumors, including TNBC, ovarian, endometrial, and HR+/HER2- BC, to further refine combination strategies.

Collectively, these trials aim to establish SG-based combinations as a cornerstone of therapy in both breast and gynecologic malignancies^[71]^.

### Dato-DXd combinatory therapies

Current clinical trials are exploring the use of Dato-DXd in combination regimens designed to enhance antitumor efficacy and broaden its therapeutic reach. Several ongoing studies are investigating combinations with immune checkpoint inhibitors to potentiate immune-mediated cytotoxicity and extend responses in TNBC and other solid tumors. For instance, the BEGONIA trial (NCT03742102), a phase Ib/II study in metastatic TNBC, evaluates Dato-DXd with durvalumab, either alone or in combination with durvalumab and paclitaxel, in TROP2- and PD-L1+ tumors^[72]^. Similarly, the TROPION-Breast03 trial (NCT05629585), a phase III study, is assessing Dato-DXd alone *vs*. Dato-DXd combined with durvalumab or TPC in patients with stage I-III TNBC who have residual disease after neoadjuvant anthracycline and taxane therapy^[73]^. The TROPION-Breast05 trial (NCT06103864) further explores whether Dato-DXd or Dato-DXd plus durvalumab can improve outcomes compared with standard chemotherapy plus pembrolizumab in metastatic TNBC^[74]^. Likewise, the TROPION-Breast04 trial (NCT06112379) compares Dato-DXd with durvalumab against pembrolizumab plus chemotherapy in TROP2- and PD-L1+ metastatic TNBC^[75]^.

The TRADE-DXd study (NCT06533826), a phase II trial, introduces a sequential treatment strategy using Dato-DXd followed by trastuzumab deruxtecan (T-DXd), or vice versa, in refractory BC. This design aims to characterize how switching between ADCs with related payload mechanisms affects outcomes and to guide optimal sequencing in practice.

In ovarian carcinoma, the DESTINITY-Ovarian01 trial (NCT06819007), a phase III study, is evaluating Dato-DXd combined with the anti-angiogenic agent bevacizumab *vs*. bevacizumab alone as first-line maintenance therapy in HER2-expressing advanced high-grade ovarian carcinoma. By targeting angiogenesis in conjunction with TROP2-directed cytotoxicity, this approach seeks to improve disease control after initial platinum-based therapy.

Additional studies focus on DNA repair pathways to maximize the impact of the topoisomerase I inhibitor payload. For example, NCT05417594 is a phase I/II trial evaluating the PARP inhibitor AZD9574 alone or in combination with Dato-DXd in advanced solid tumors, including ovarian, breast, pancreatic, and prostate cancers. Similarly, NCT04644068 examines AZD5305, another PARP inhibitor, alone or with Dato-DXd across multiple tumor types such as ovarian, breast, pancreatic, prostate, colorectal, gastric, bladder, cervical, and endometrial cancers.

Collectively, these trials aim to integrate Dato-DXd into rational combination and sequencing strategies across diverse solid malignancies.

### SAC-TMT combinatory therapies and resistance modulation

Sac-TMT is under active investigation in combination regimens intended to enhance efficacy across gynecologic malignancies. One phase II trial, NCT05642780, is evaluating Sac-TMT together with pembrolizumab in previously treated patients with cervical cancer. This combination seeks to harness both TROP2-directed cytotoxicity and immune checkpoint blockade to improve outcomes in a population with limited options^[76]^. In parallel, the phase III trial MK-2870-022/TroFuse-022/GOG-3103/ENGOT-ov84 (NCT06824467) is assessing Sac-TMT alone or in combination with bevacizumab *vs*. the TPC in patients with platinum-sensitive recurrent ovarian carcinoma. By co-targeting TROP2 and angiogenesis, this study aims to maximize disease control in the recurrent setting. Together, these trials underscore the expanding role of Sac-TMT–based combination strategies in recurrent and treatment-refractory gynecologic cancers.

## MECHANISMS OF RESISTANCE TO TROP2-DIRECTED ADCS AND POTENTIAL BIOMARKERS

This section examines the molecular mechanisms of resistance to TROP2-direct ADCs. Elucidating those mechanisms provides a critical framework for biomarker discovery and for optimizing patient selection and improving patient outcomes [Figure 1].

### Antigen expression and ADC internalization

Primary and acquired resistance to TROP2-directed ADCs can arise from loss or downregulation of TROP2, as well as heterogeneous antigen expression across tumor cell populations, which together reduce effective target engagement and payload delivery. Decreased or uneven TROP2 expression has been associated with limited response duration and relapse after initial benefit with SG in TNBC and in other solid tumors. Impaired ADC internalization following antigen binding, or defects in intracellular trafficking to lysosomes, can further compromise payload release and lower cytotoxic activity, affecting agents such as SG, Dato-DXd, and Sac-TMT^[43,44,60]^.

### Payload efflux and intracellular drug handling

Upregulation of efflux transporters, including ABC family pumps such as ABCG2, can promote active export of topoisomerase I inhibitor payloads out of tumor cells, thereby reducing intracellular accumulation of SN-38, DXd, or related compounds and weakening their cytotoxic effects^[43]^. Alterations in lysosomal processing, linker cleavage, or intracellular stability of the released drug can similarly diminish effective payload concentration at its nuclear target, contributing to both intrinsic non-response and secondary resistance to SG and Dato-DXd. In ovarian and gynecologic tumors treated with SG or Sac-TMT, increased efflux pump activity and suboptimal intracellular drug handling have been implicated in diminished treatment durability^[45,60]^.

### DNA damage repair and cell-cycle adaptation

Enhanced DNA damage repair capacity is a central mechanism through which tumors adapt to topoisomerase I inhibitor–based ADCs. Upregulation or reactivation of repair pathways can counterbalance the DNA strand breaks induced by SN-38, DXd, or KL610023, thereby permitting survival despite continued drug exposure. In this context, the tumor genetic background, particularly alterations in BRCA1/2 and defects in homologous recombination (HR) repair, plays a pivotal role in modulating sensitivity to TROP2-directed ADCs. BRCA-mutated or HR-deficient tumors exhibit impaired repair of replication-associated DNA damage, rendering them more vulnerable to induced cytotoxicity. Adaptive changes in cell-cycle regulation, including shifts that favor enhanced repair during specific cell-cycle phases, also facilitate escape from ADC-induced DNA damage and have been proposed to underlie resistance-associated relapse in TNBC and other solid malignancies. These repair and cell-cycle–driven adaptations help explain cross-resistance patterns observed when sequencing different topoisomerase I–based ADCs such as SG, Dato-DXd, and Sac-TMT^[44,52,54,60,71]^.

### Pro-survival signaling and apoptosis escape

Activation of pro-survival signaling pathways can blunt the pro-apoptotic effects of topoisomerase I–mediated DNA damage and contribute to resistance to TROP2-directed ADCs. Oncogenic signaling networks that promote cell survival, as well as specific anti-apoptotic mechanisms, can mitigate the cytotoxic impact of SG, Dato-DXd, and Sac-TMT even in the presence of adequate payload delivery. These adaptations underpin efforts to combine TROP2-directed ADCs with agents targeting apoptotic regulators, such as MCL-1 inhibitors, or with cell-cycle modulators, to re-sensitize resistant tumor cells and restore ADC efficacy^[44]^.

### Tumor microenvironment, hypoxia, and drug penetration

The tumor microenvironment also plays a key role in limiting the activity of TROP2-directed ADCs. Structural features such as dense stroma, elevated interstitial pressure, and abnormal vasculature can restrict ADC penetration and distribution within tumor tissue, favoring survival of poorly exposed cancer cell subclones. Hypoxia and angiogenic remodeling further impair drug delivery and create niches that support resistant clones, which is particularly relevant in ovarian and endometrial cancers where anti-angiogenic combinations with Dato-DXd or Sac-TMT aim to overcome these barriers. Tumor microenvironment can also modulate antigen expression through cytokine signaling, hypoxia, or extracellular matrix interactions. An immunosuppressive microenvironment can additionally dampen immune-mediated components of ADC activity, facilitating immune escape and treatment failure^[45,65]^.

### Immune evasion and resistance to immune–ADC combinations

Immune evasion mechanisms, including adaptive upregulation of immune checkpoints and recruitment of immunosuppressive cells, can limit the benefit of combining TROP2-directed ADCs with immune checkpoint inhibitors. Tumors that develop or maintain an immunologically “cold” microenvironment may fail to mount sufficient antitumor immune responses despite ADC-induced immunogenic cell death, thereby sustaining resistance. These observations have motivated multiple clinical trials combining SG, Dato-DXd, or Sac-TMT with PD-1/PD-L1 inhibitors and other immunomodulatory agents to convert resistant tumors into more inflamed states and to counteract checkpoint-mediated adaptive resistance^[65,75]^.

### Cross-resistance and sequencing of ADCs

Use of multiple topoisomerase I–based ADCs across the disease course raises the issue of cross-resistance driven by shared payload mechanisms. Prior exposure to SG or other DNA-damaging agents may prime tumors to upregulate DNA repair, efflux transporters, and survival pathways, potentially reducing sensitivity to subsequent Dato-DXd or Sac-TMT. Emerging sequential-trial designs are therefore exploring how order and timing of different ADCs influence resistance development, with the aim of identifying treatment sequences that delay or minimize cross-resistance and maximize long-term benefit.

## DISCUSSION

The emergence of ADCs targeting TROP2 has marked a significant advancement in the therapeutic landscape for aggressive malignancies such as TNBC and ovarian carcinoma. This review highlights both the clinical efficacy and mechanistic rationale underlying TROP2-directed ADCs, particularly SG, Dato-DXd, and Sac-TMT. The growing body of evidence confirms that TROP2 overexpression drives tumor proliferation, invasion, and metastasis, correlating strongly with poor clinical outcomes in TNBC and ovarian carcinoma. The clinical success of SG, demonstrated in the ASCENT trial, has established a proof of concept for targeting TROP2 in metastatic TNBC, with significant improvements in ORR, PFS, and OS. Similarly, Dato-DXd has shown encouraging activity in heavily pretreated TNBC and is being actively investigated in ovarian carcinoma.

Hematologic and gastrointestinal adverse events represent the most common toxicities and require proactive monitoring and intervention. Early identification and management of neutropenia through routine blood count surveillance, timely dose modifications, and the selective use of granulocyte colony-stimulating factor are essential to prevent treatment delays and infectious complications. Diarrhea and other gastrointestinal toxicities should be addressed through prompt initiation of antidiarrheal agents, adequate hydration. Dato-DXd is associated with stomatitis, nausea, and fatigue, which can be effectively managed through prophylactic oral care protocols, topical corticosteroids, and supportive antiemetic strategies. Early intervention and temporary dose interruptions are particularly important to prevent escalation to higher-grade toxicity and to maintain long-term treatment adherence.

SG uses an SN-38 payload with a relatively hydrolysable linker and high DAR, Dato-DXd uses the DXd payload with a more plasma-stable tetrapeptide linker and DAR 4, and Sac-TMT couples the same anti-TROP2 antibody as SG to a belotecan-derived payload via a novel, more stable cleavable linker. Among these agents, SG is currently the ADC with the most clearly characterized clinical resistance, with genomic studies describing TOP1 mutations that reduce SN-38 sensitivity and a TROP2 (TACSTD2) T256R mutation that impairs membrane localization and antibody binding. Patients who benefit most from SG are those with pre-treated metastatic TNBC or HR+/HER2- BC and preserved TROP2 expression, while Dato-DXd is emerging for TROP2-high epithelial tumors such as BC and NSCLC in need of an improved safety–efficacy balance, and Sac-TMT shows particular promise in heavily pre-treated TNBC and EGFR-mutated NSCLC after tyrosine kinase inhibitors. Resistance mechanisms are partly shared, including downregulation or mutation of TROP2 that limits ADC binding and internalization, and alterations in topoisomerase I or DNA-damage response that decrease sensitivity to topo-I payloads across the class. However, they also diverge in a payload- and linkerdependent manner, so tumors that acquire resistance to SG through SN38specific TOP1 alterations or through handling of its less stable linker may retain some sensitivity to Dato-DXd or Sac-TMT, which use different topoI warheads and more stable linker designs.

Resistance remains a central challenge limiting the long-term efficacy of TROP2-targeted ADCs. Key mechanisms include antigen downregulation or loss, heterogeneous TROP2 expression, impaired internalization and trafficking, drug efflux via ABC transporters, and lysosomal dysfunction, all of which reduce intracellular payload delivery and cytotoxicity. Additionally, tumor microenvironmental factors, such as stromal barriers, hypoxia, and immunosuppressive signaling, further restrict ADC penetration and contribute to adaptive resistance. To overcome these limitations, innovative therapeutic strategies are actively being developed. A thorough understanding of the molecular and cellular mechanisms underlying resistance is essential for the rational design of effective treatment approaches and for improving clinical outcomes. Next-generation ADCs, including bispecific constructs, dual-payload platforms, and linker–payload optimizations, are designed to enhance tumor selectivity, bypass efflux mechanisms, and broaden cytotoxic effects. Moreover, rational combination regimens with immune checkpoint inhibitors, PARP inhibitors, and anti-angiogenic agents aim to synergistically counteract resistance pathways and potentiate immune-mediated tumor clearance. Emerging candidates, such as DB-1305/BNT325, exemplify this evolution, integrating enhanced linker stability and payload potency to overcome acquired resistance. This next-generation anti-TROP2 ADC, currently in Phase I/IIa clinical evaluation (NCT05438329), has received FDA fast track designation for the treatment of platinum-resistant ovarian, fallopian tube, and primary peritoneal carcinomas, highlighting its potential to address unmet clinical needs in therapy-refractory populations.

Other major challenges remain in identifying robust predictive biomarkers to optimize patient selection and in establishing durable efficacy and safety across diverse clinical settings [Figure 1]. Although TROP2 expression is required for patient eligibility, it is insufficient to reliably predict response to TROP2-directed ADCs, highlighting the need for integrated, multidimensional biomarker strategies. Future research should continue to elucidate the molecular and cellular mechanisms underlying ADC resistance, thereby enabling the rational development of combination approaches and next-generation ADC platforms.

Genomic profiling can identify alterations in DNA damage response pathways, such as BRCA1/2 mutations and HR deficiency, which may predict sensitivity to topoisomerase I–based payloads or inform rational combination strategies. Transcriptomic analyses provide insight into dynamic regulation of TROP2 expression and pathways involved in intracellular trafficking, lysosomal function, drug efflux, and cell-cycle control. Proteomic approaches further refine patient stratification by directly assessing TROP2 protein levels, payload target expression, and activation of survival or apoptotic pathways, as well as tumor-stroma interactions. Finally, spatial pathology-based techniques preserve tissue context and reveal intratumoral heterogeneity in antigen expression, immune infiltration, and stromal architecture, all of which critically influence ADC penetration and therapeutic efficacy.

In summary, the development of TROP2-targeted ADCs represents a paradigm shift in the management of TNBC and ovarian carcinoma, offering new hope for patients with limited therapeutic options. The encouraging clinical outcomes observed thus far, together with ongoing efforts to address resistance and expand the applicability of these agents, suggest that ADCs will play an increasingly prominent role in the future of precision oncology. Ongoing investigation into the molecular mechanisms underlying therapeutic response, along with the strategic development of next-generation ADCs, will be crucial to unlocking the full therapeutic potential of this drug class.

## DECLARATIONS

### Copyright

© The Author(s) 2026.
